# Efficient depolymerization of lignin through microwave-assisted Ru/C catalyst cooperated with metal chloride in methanol/formic acid media

**DOI:** 10.3389/fbioe.2022.1082341

**Published:** 2022-12-16

**Authors:** Lupeng Shao, Chao Wang, Yu Liu, Meng Wang, Luyan Wang, Feng Xu

**Affiliations:** ^1^ State Key Laboratory of Biobased Material and Green Papermaking, Key Laboratory of Pulp and Paper Science & Technology (Ministry of Education), Qilu University of Technology (Shandong Academy of Sciences), Jinan, China; ^2^ Shandong Chenming Paper Holdings Co., Ltd., Weifang, China; ^3^ Beijing Key Laboratory of Lignocellulosic Chemistry, Beijing Forestry University, Beijing, China

**Keywords:** lignin, microwave, degradation, synergetic catalysis, controllable product distribution

## Abstract

Lignin, an abundant aromatic biopolymer, has the potential to produce various biofuels and chemicals through biorefinery activities and is expected to benefit the future circular economy. Microwave-assisted efficient degradation of lignin in methanol/formic acid over Ru/C catalyst cooperated with metal chloride was investigated, concerning the effect of type and dosage of metal chloride, dosage of Ru/C, reaction temperature, and reaction time on depolymerized product yield and distribution. Results showed that 91.1 wt% yield of bio-oil including 13.4 wt% monomers was obtained under the optimum condition. Yields of guaiacol-type compounds and 2,3-dihydrobenzofuran were promoted in the presence of ZnCl_2_. Formic acid played two roles: (1) acid-catalyzed cleavage of linkages; (2) acted as an *in situ* hydrogen donor for hydrodeoxygenation in the presence of Ru/C. A possible mechanism for lignin degradation was proposed. This work will provide a beneficial approach for efficient depolymerization of lignin and controllable product distribution.

## 1 Introduction

The global population growth and the development of industrialization lead to a rising demand for fuels and chemicals, resulting in many societal problems, such as energy security and environmental concerns (Sun et al., 2018; Wang et al., 2019; Jing et al., 2020; Ma et al., 2021). Developing renewable and environment-friendly energy has become an essential measure for many countries to promote energy transformation. Lignin, which constitutes up to 40% of the energy content of most terrestrial plants, shows great potential to produce liquid fuel or petroleum-based aromatic chemicals owing to its aromatic building blocks (Regalbuto, 2009; Barta et al., 2010; Cao et al., 2019; Ekielski and Mishra, 2020; Tang et al., 2022; Zhou et al., 2022).

Despite various challenges associated with lignin valorization, several strategies have emerged that could deliver value-added products, such as biological (Chong et al., 2018) and chemical process (Li et al., 2015). In particular, a wide range of thermochemical approaches for converting lignin into aromatic-rich bio-oil, including pyrolysis (Shao et al., 2017; Cao et al., 2021), acid/base catalytic hydrolysis (Deuss et al., 2015; Hernández-Ramos et al., 2020), oxidation (Lancefield et al., 2015; Sun et al., 2020) and reductive depolymerization (Liu et al., 2019), have been extensively investigated. Bio-oil is a higher energy content transportable liquid for subsequent production of biofuels and aromatic chemicals. Previous researches have indicated that solvents and catalysts have significant effects on lignin depolymerization. Various solvents (such as water, alcohol, formic acid, etc.) were investigated during the lignin valorization process. Shu et al. (2016b) investigated the influence of solvent on lignin depolymerization, and concluded that supercritical methanol showed the bested depolymerization performance. Hidajat et al. (2018) used formic acid as hydrogen source to produce oxygen-free aromatics, achieving high-calorific-value oil (> 40 MJ kg−1) with a high content of oxygen-free aromatics (6.8 wt%). Various kinds of catalysts, including homogeneous and heterogeneous catalysts, have been extensively tested (Wang et al., 2019). Kristianto et al. (2017) reported the depolymerization of concentrated acid hydrolysis lignin using a Ru/C catalyst in ethanol/formic acid media, achieving a high bio-oil yield of ∼70 wt% and a high heating value of 32.7 MJ kg^−1^. Luo et al. (2020) used boric acid as a novel homogeneous catalyst coupled with Ru/C to overcome the product separation problem and perform the hydrodeoxygenation (HDO) of phenolic compounds and raw lignin oil. The content of hydrocarbons increases from 7.9% to 93.1% at 260°C. Most of these thermochemical approaches required relatively harsh reaction conditions (high reaction temperature and/or high pressure). Therefore, it is urgent to explore mild reaction conditions and technologies for converting lignin into high-value liquid fuel and/or aromatic chemicals.

Recently, in comparison with those conventional heating processes, microwave-assisted depolymerization of lignin has attracted increasing attention on account of its advantages of fast heating rate and high heating efficiency (Liu et al., 2017; Zhou et al., 2019; Cederholm et al., 2020). Microwave heating occurs *via* dipole rotation and ionic conduction. Therefore, polar solvents which have high microwave absorption abilities are adopted as additional microwave receptors. Moreover, the addition of molecular hydrogen will increase the cost and risk of the process (Jiang et al., 2019). Consequently, the use of polar hydrogen-donating reagents (methanol, ethanol, formic acid, et al.) would be potentially safer and more cost-effective (Zhou et al., 2018). Toledano et al. investigated the influence of various hydrogen-donating solvents on lignin depolymerization under microwave conditions, finding that formic acid showed the best performance (Toledano et al., 2013). Apart from solvents, catalysts also played an essential role in the microwave-assisted depolymerization of lignin. Noble metal-based catalysts (such as Pd, Pt, and Ru, etc.) were proved to be effective for lignin depolymerization (Zhang et al., 2019; Wang et al., 2021). Previous studies indicated that the combination of the noble metal with acid catalyst had better performances for lignin depolymerization (Klein et al., 2016; Bjelić et al., 2020; Jiang et al., 2021). Shu et al. investigated the catalytic performances of various metal chlorides cooperated with Pd/C for lignin depolymerization and observed that the highest yield (28.5 wt%) of phenolic monomers was obtained with CrCl_3_ under harsh conditions (4 MPa H_2_, 260°C, 5 h) (Shu et al., 2015).

In our previous work, microwave-assisted degradation of alkaline lignin in methanol/formic acid (FA) media was investigated, and 72.0 wt% yield of bio-oil including 6.7 wt% monomers was achieved at 160°C and a FA-to-lignin mass ratio of 4 after a reaction time of 30 min (Shao et al., 2018). Herein, highly efficient depolymerization of lignin in methanol/formic acid over Ru/C catalyst cooperated with metal chlorides assisted by microwave heating is reported. The effects of catalyst, reaction temperature, and reaction time on the product yield and distribution are studied in detail. The possible mechanism of lignin depolymerization is proposed.

## 2 Materials and methods

### 2.1 Materials

The alkaline lignin was provided by Shandong Longlive Bio-Technology Co., Ltd., China, a by-product of xylooligosaccharides production from corn cob. The lignin was composed of 90.81% Klason lignin, 3.61% acid-soluble lignin, 0.63% sugars, 2.16% ash, and 2.79% others (Shao et al., 2018). 5 wt% Ru/C, formic acid (FA, 99%), ethyl acetate (HPLC grade, 99.9%), and acetophenone (standard for GC, 99.5%) were provided by Aladdin^®^ company. Methanol (99.5%), ethyl acetate (99.5%), and tetrahydrofuran (THF, 99.0%) were analytical grade and provided by Beijing Chemical Works. AlCl_3_, CrCl_3_, LiCl, NaCl, FeCl_3_, FeCl_2_, MgCl_2_, ZnCl_2_, KCl, and Zn(OAC)_2_ were also analytical grade and purchased from Macklin^®^ company. All reagents were used as received.

### 2.2 Lignin depolymerization

Microwave-assisted depolymerization of lignin was carried out in microwave digestion instrument (MDS-6G, Sineo Microwave Technology Co., Ltd., China). Methanol and formic acid (FA) were chosen as solvent and hydrogen donor during the depolymerization process. Ru/C and metal chloride were used as catalysts. For a typical experiment, 1 g lignin, 1 mmol metal chloride, 0.2 g 5 wt% Ru/C, 20 ml methanol, and 4 g formic acid (FA) were added into the digestion tank. The reaction was conducted at 400 W under settled reaction temperature (140, 160, and 180°C) and reaction time (15, 30, and 45 min). After the reaction, the mixture was cooled down to ambient temperature.

### 2.3 Products separation and analysis

The products separation was as follows. The reaction mixture was first filtered, and the filter cake was washed three times with methanol. The solid fraction 1 (including catalyst and residue) was obtained. The filtrate was collected and subjected to rotary evaporation, followed by adding ethyl acetate. The resulting mixture was filtered again. The solid fraction 2 was obtained. The filtrate was rotary evaporated, achieving the bio-oil. The solid fractions were washed with THF and filtered, achieving the residue and catalyst. The yields of bio-oil, aromatic monomers, and residue were calculated according to the following equations:

$$Y_{AM}\left(\%\right)=Yield\;of\;aromatic\;monomer=W_{AM}/W_{L}\times 100\%$$ (1)

$$Y_{BO}\left(\%\right)=Yield\;of\;bio-oil=W_{Bo}/W_{L}\times 100\%$$ (2)

$$Y_{R}\left(\%\right)=Yield\;of\;residue=W_{R}/W_{L}\times 100\%$$ (3)

Where W_AM_, W_BO_, W_R_, and W_L_ depict the weight of aromatic monomer, bio-oil, residue, and starting lignin, respectively.

Qualitative and quantitative analysis of aromatic monomers in bio-oil were carried out by SHIMADZU GC–MS-QP 2010 SE and SHIMADZU GC-2010 Plus with a FID detector equipped with HP-5 capillary columns (30 m × 0.25 mm × 0.25 μm), respectively. The oven temperature was programmed from 50°C to 300°C with a 10°C /min heating rate. The injector was kept at 250°C with a split ratio of 20, using helium as carrier gas. Acetophenone was used as the internal standard.

## 3 Results and discussion

### 3.1 Product distribution of lignin depolymerization

#### 3.1.1 Effect of metal chloride

Various products can be obtained after lignin depolymerization. Table 1 shows the effect of different metal chloride on bio-oil, aromatic monomer, and residue yields. The specific aromatic monomer distribution and yield are shown in Supplementary Table S1. Generally, the distribution of depolymerized lignin products was complex. According to the specific functional groups, products were classified into four categories: 2,3-dihydrobenzofuran (DHBF), phenol-type compounds (H), guaiacol-type compounds (G), and syringol-type compounds (S). It should be noted that no chloride-contained compounds were detected in products, indicating that dissolved chloride did not react with the lignin.

**TABLE 1:** The effect of different metal chloride on the products of lignin depolymerization.

Entry	Metal chloride	Y_BO_ (%)	Y_AM_ (%)	Y_R_ (%)
1	-^a^	72.0	6.7	20
2	-^b^	74.2	5.4	24.6
3	ZnCl_2_	84.3	10.2	17.3
4	AlCl_3_	69.3	5.4	29.6
5	CrCl_3_	78.8	9.0	18.9
6	LiCl	63.6	5.0	35.7
7	FeCl_3_	74.5	7.5	24.6
8	NaCl	68.6	6.0	29.2
9	KCl	65.8	5.6	33.6
10	FeCl_2_	70.2	6.8	31.8
11	MgCl_2_	71.7	7.5	34.8
12	Zn(OAC)_2_	69.2	6.9	32.1

^a^ without both metal chloride and Ru/C.

^b^ without metal chloride.

Y_BO_: Yield of bio-oil, Y_AM_: Yield of aromatic monomer, Y_R_: Yield of residue.

Condition: 1 g lignin, 1 mmol metal chloride, 0.2 g 5 wt% Ru/C, 20 ml methanol, 4 g formic acid, 160°C, 30 min.

As shown in Table 1, the bio-oil yield increased slightly after adding Ru/C catalyst, but the promotion effect on the aromatic monomer yield was not significant. The yield of bio-oil increased or decreased when metal chloride was added. It is generally known that Cl is a high electronegativity element, and Cl^−1^ was widely used in biomass dissolution and conversion as an excellent nucleophilic regent (Long et al., 2011; Shu et al., 2015). C-O bond in cellulose was easier to be broken in most cellulose conversion reactions using chloride ionic liquids as solvent (Swatloski et al., 2002). Lignin contained a large number of ether bonds, especially the β-O-4′ bonds. Therefore, it is considered that Cl^−1^ in metal chloride can work in lignin depolymerization. Data in Table 1 showed that ZnCl_2_, CrCl_3_, and FeCl_3_ significantly promoted lignin depolymerization. The common feature was the higher valence of metal cation. The result showed that metal cations also affected lignin depolymerization, which may be related to the Lewis acid strength of metal cation. It has been reported that metal cations can produce more acid centers (Miessler et al., 2013), promoting lignin depolymerization. One more interesting phenomenon, metal cations of AlCl_3_ and MgCl_2_ also had high valence, but the bio-oil yield was lower than that of other high-valent metal cations. This might be caused by hydrated metal chloride used in the experiment, such as AlCl_3_•6H_2_O and MgCl_2_•6H_2_O. Water present in the reagents might affect lignin depolymerization, which was also confirmed by the high residue yield.

Yields of bio-oil and aromatic monomers were the highest in the presence of ZnCl_2_. The result differed from Shu et al. (2018)’s research, who believed that CrCl_3_ provided a better catalytic effect. The higher valence of Cr^3+^ in CrCl_3_ could produce more acidic centers, which promoted lignin depolymerization. Cr^3+^ was first conjunct with O and benzene ring of lignin molecule to form a stable complex. Synchronously, Cl^−^ with high electronegativity was connected to lignin molecule, resulting in the breakage of C-O bonds (Tang et al., 2021). Furthermore, hydrogenolysis was promoted by capturing hydrogen under the polarization effect of Cl^−^ and the synergic effect of Pd/C (Parsell et al., 2013). The product distribution was significantly different by comparing the aromatic monomers generated from ZnCl_2_ and CrCl_3_ synergistic Ru/C catalytic depolymerization of lignin. For example, when ZnCl_2_ was used, the yield of 4-ethylphenol (H2, 0.89 wt%) was the highest among the H-type compounds, without the generation of *p*-coumaric acid (H4). When CrCl_3_ was used, it showed the opposite result that the yield of H4 (1.55 wt%) was the highest among the H-type compounds without H2. The result indicated that the catalytic mechanism of ZnCl_2_ and CrCl_3_ was different. Compared with other metal chloride, using ZnCl_2_ could facilitate the formation of H4 and increase the yield of G-type compounds. Besides, the highest yield (5.58 wt%) of 2,3-dihydrobenzofuran (DHBF) was obtained. Significant decreases of bio-oil and total aromatic monomers were exhibited when ZnCl_2_ was replaced by Zn(OAc)_2_. Meanwhile, a dramatic change of product distribution occurred, especially the S-type compounds. It indicated that the synergic effect between Zn^2+^ and Cl^−^ was contributed to improve the yields of bio-oil and aromatic monomer. Data showed that lignin could be efficiently depolymerized under the catalyst of ZnCl_2_ cooperated with Ru/C.

#### 3.1.2 Effect of ZnCl_2_ dosage

The effect of ZnCl_2_ dosage on lignin depolymerization was investigated. As shown in Table 2, the category and yield of S-type compounds decreased with the increase of ZnCl_2_ dosage. Only S3 was produced when the dosage of ZnCl_2_ was 1 and 2 mmol. As the dosage of ZnCl_2_ was further increased to 3 mmol, no S-type compounds were produced. In view of the lower yield of S-type compounds in depolymerized products, yields of bio-oil, total monomers, DHBF, H-type, and G-type compounds were mainly discussed as follows.

**TABLE 2:** Effect of ZnCl_2_ dosage on the distribution of lignin-derived aromatic monomers.

Type^a^	Compound	Retention time (min)	Yield (%)				
			ZnCl_2_ dosage (mmol)				
			0	0.5	1	2	3
DHBF	2,3-Dihydrobenzofuran	13.832	2.57	3.63	5.6	2.48	2.03
H1	Phenol	8.731				1.26	1.46
H2	Phenol, 4-ethyl-	12.753		0.23	0.89	2.47	3.11
H3	2-Propenoic acid, 3-(4-hydroxyphenyl)-, methyl ester	22.837	0.12	0.29	0.44	0.66	1.06
H4	*p*-Coumaric acid	23.356	1.18	1.91			
G1	Phenol, 2-methoxy-	11.109				0.91	1.1
G2	Phenol, 4-ethyl-2-methoxy-	14.999		0.08	0.25	0.5	0.75
G3	Phenol, 2-methoxy-4-vinyl-	15.679		0.57	0.98	0.66	0.29
G4	Vanillin	17.293	0.23	0.29	0.33	0.52	0.49
G5	2-Propenoic acid, 3-(4-hydroxy-3-methoxyphenyl)-, methyl ester	24.544	0.47	0.39	0.85	1.11	
G6	2-Propenoic acid, 3-(4-hydroxy-3-methoxyphenyl)-	24.763	0.37	0.27	0.6	2.7	1.29
S1	Benzaldehyde, 4-hydroxy-3,5-dimethoxy-	21.572	0.08	0.17			
S2	Phenol, 2,6-dimethoxy-4-propenyl-	22.136	0.15	0.07			
S3	Ethanone, 1-(4-hydroxy-3,5-dimethoxyphenyl)-	22.668	0.13	0.15	0.24	0.17	
S4	3,5-Dimethoxy-4-hydroxycinnamaldehyde	26.137	0.08				

^a^ DHBF: 2,3-dihydrobenzofuran, H: phenol-type compounds, G: guaiacol-type compounds, S: syringol-type compounds.

Condition: 1 g lignin, 0.2 g 5 wt% Ru/C, 20 ml methanol, 4 g formic acid, 160°C, 30 min.

**FIGURE 1:**
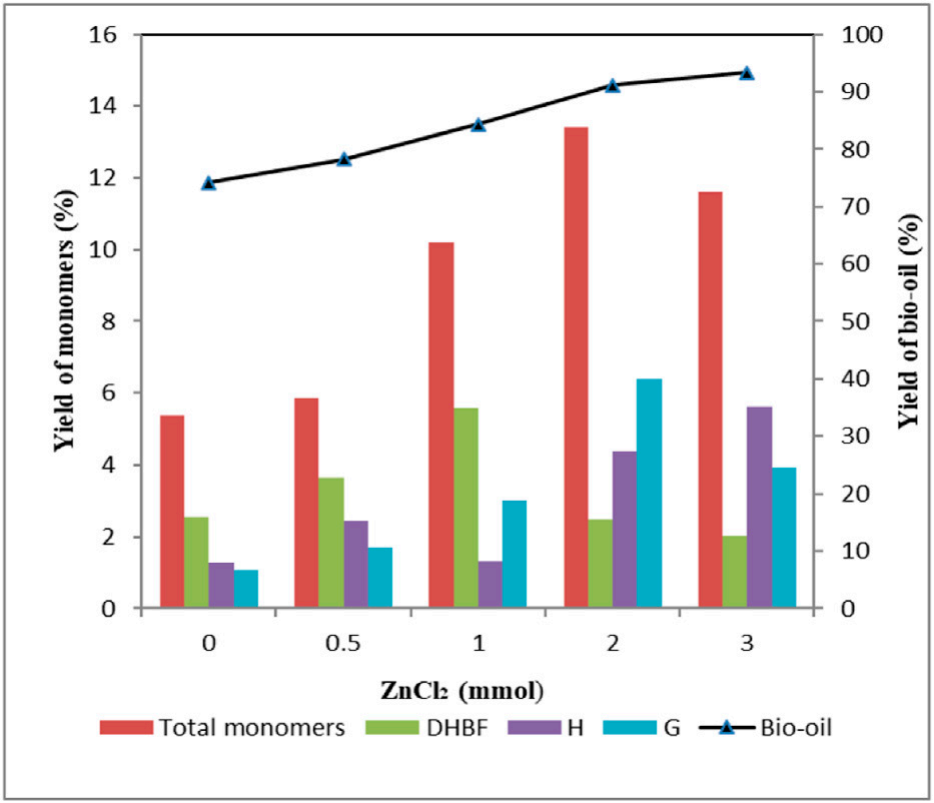
Effect of ZnCl_2_ dosage on the yield of depolymerized products. Condition: 1 g lignin, 0.2 g 5 wt% Ru/C, 20 ml methanol, 4 g formic acid, 160°C, 30 min.

As shown in Figure 1, the yield of bio-oil increased with increasing ZnCl_2_ dosage. And the highest bio-oil yield of 93.4 wt% was obtained when 3 mmol ZnCl_2_ was used. The yield of total monomers rose from 5.38 wt% to 13.4 wt% with increasing ZnCl_2_ dosage from 0 to 2 mmol. However, as ZnCl_2_ dosage was further increased to 3 mmol, yield of total monomers decreased to 11.6 wt%. This phenomenon suggested that lignin depolymerization was favored at high ZnCl_2_ dosage; meanwhile, repolymerization of monomers to form oligomers was also promoted. Among the monomers, DHBF was abundantly produced; the yield first increased to 5.6 wt% and then decreased to 2.03 wt% as a function of ZnCl_2_ dosage. The yield of H-type compounds increased as the amount of ZnCl_2_ increased, and the highest yield (5.63 wt%) was obtained when 3 mmol ZnCl_2_ was used. It should be noted that H2 was produced only in the presence of ZnCl_2_, and phenol was produced only when the ZnCl_2_ dosage was more than 2 mmol. The yields of H2 and phenol were proportional to ZnCl_2_ dosage. This phenomenon indicated that increasing the dosage of ZnCl_2_ was conductive to the formation of H1 and H2. The highest yield (1.91 wt%) of *p*-coumaric acid appeared when the ZnCl_2_ dosage was 0.5 mmol. Further increasing ZnCl_2_ dosage, no *p*-coumaric acid was detected in products. This indicated that high ZnCl_2_ dosage inhibited the production of *p*-coumaric acid. For G-type compounds, the yield first increased to 6.4 wt% and then decreased to 3.92 wt% as a function of ZnCl_2_ dosage. The decrease in the yield of G-type compounds was mainly caused by a decline of G3, G4, G5, and G6. The yields of G1 and G2 showed similar variation tendency with H1 and H2, respectively, when increasing ZnCl_2_ dosage. The result indicated great significance for controlling the selectivity of depolymerized products by varying ZnCl_2_ dosage.

#### 3.1.3 Effect of Ru/C dosage

**FIGURE 2:**
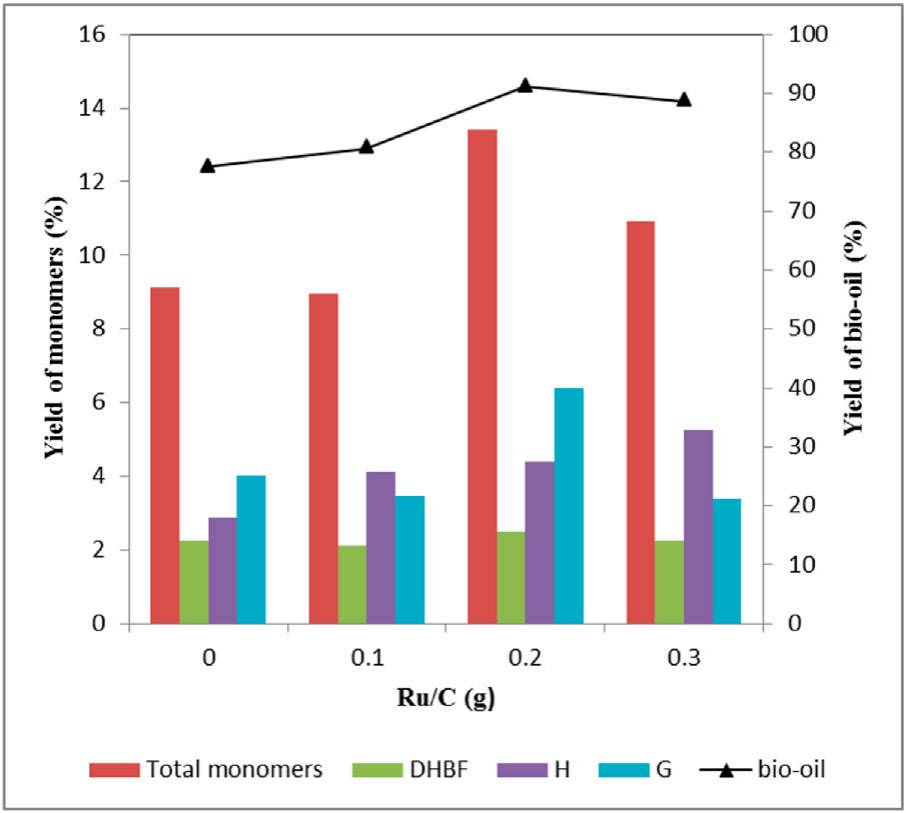
Effect of Ru/C dosage on the yield of depolymerized products Condition: 1 g lignin, 2 mmol ZnCl_2_, 20 ml methanol, 4 g formic acid, 160°C, 30 min.

The effect of Ru/C dosage had also been carefully examined. Table 3 shows the distribution of aromatic monomers. When the amount of ZnCl_2_ was 2 mmol, there were basically no S-type phenolic compounds in the products regardless of the amount of Ru/C. As can be seen from Figure 2, the yield of bio-oil increased after adding Ru/C catalyst. The highest bio-oil yield (91.1 wt%) and total aromatic monomer yield (13.4 wt%) were obtained when 0.2 g Ru/C was used. Further adding Ru/C dosage would decrease the yields of bio-oil and total aromatic monomer. According to Figure 2, the amount of Ru/C had little effect on the yield of DHBF, but mainly affected the yield of H-type and G-type compounds. The yield of H-type compound was proportional to the amount of Ru/C, and the main products were H1 and H2. For G-type compounds, the highest yield (6.4 wt%) was obtained when 0.2 g Ru/C was used. The yield of G1 increased after the addition of Ru/C, but the amount of Ru/C had little effect on the yield, basically keeping at 0.9 wt%. The increase of Ru/C dosage was beneficial to the production of G2, but decreased the yield of G4. According to Table 4, when the amount of Ru/C increased, the yield of the products containing carbonyl group or double bonds in the side chain showed a decreasing trend. In contrast, the yield of products without unsaturated bonds in the side chain tended to increase. The result demonstrated that Ru/C facilitated hydrodeoxygenation in the side chain of benzene ring (Chen et al., 2015). The reason was that formic acid, as an *in situ* hydrogen source, could generate active hydrogen species using Ru/C catalyst under solvothermal conditions (Kristianto et al., 2017), which could facilitate hydrodeoxygenation. The role of formic acid was different from our previous study (Shao et al., 2018), in which formic acid acted through acid-catalyzed cleavage of linkages in lignin. In the presence of Ru/C, formic acid played two roles: (1) acid-catalyzed cleavage of linkages; (2) acted as an *in situ* hydrogen donor.

**TABLE 3:** Effect of Ru/C dosage on the distribution of lignin-derived aromatic monomers.

Type^a^	Compound	Retention time (min)	Yield (%)			
			Ru/C dosage (g)			
			0	0.1	0.2	0.3
DHBF	2,3-Dihydrobenzofuran	13.832	2.27	2.1	2.48	2.27
H1	Phenol	8.731	0.8	0.86	1.26	1.56
H2	Phenol, 4-ethyl-	12.753	2.07	3.26	2.47	3.7
H3	2-Propenoic acid, 3-(4-hydroxyphenyl)-, methyl ester	22.837			0.66	
G1	Phenol, 2-methoxy-	11.109	0.6	0.9	0.91	0.88
G2	Phenol, 4-ethyl-2-methoxy-	14.999	0.48	0.64	0.5	0.81
G3	Phenol, 2-methoxy-4-vinyl-	15.679	0.6	0.22	0.66	
G4	Vanillin	17.293	0.71	0.45	0.52	0.47
G5	2-Propenoic acid, 3-(4-hydroxy-3-methoxyphenyl)-, methyl ester	24.544	0.57		1.11	
G6	2-Propenoic acid,3-(4-hydroxy-3-methoxyphenyl)-	24.763	1.04	1.27	2.7	1.22
S3	Ethanone, 1-(4-hydroxy-3,5-dimethoxyphenyl)-	22.668			0.17	

^a^ :DHBF: 2,3-dihydrobenzofuran, H: phenol-type compounds, G: guaiacol-type compounds, S: syringol-type compounds.

Condition: 1 g lignin, 2 mmol ZnCl_2_, 20 ml methanol, 4 g formic acid, 160°C, 30 min.

**TABLE 4:** Effect of reaction temperature on the distribution of lignin-derived aromatic monomers.

Type^a^	Compound	Retention time (min)	Yield (%)		
			Reaction temperature (^o^C)		
			140	160	180
DHBF	2,3-Dihydrobenzofuran	13.832	1.64	2.48	1.45
H1	Phenol	8.731	1.14	1.26	1.44
H2	Phenol, 4-ethyl-	12.753	2.34	2.47	2.64
H3	2-Propenoic acid, 3-(4-hydroxyphenyl)-, methyl ester methyl ester	22.837		0.66	0.89
G1	Phenol, 2-methoxy-	11.109	0.76	0.91	0.88
G2	Phenol, 4-ethyl-2-methoxy-	14.999	0.42	0.5	0.66
G3	Phenol, 2-methoxy-4-vinyl-	15.679	0.41	0.66	0.2
G4	Vanillin	17.293		0.52	
G5	2-Propenoic acid, 3-(4-hydroxy-3-methoxyphenyl)-, methyl ester	24.544	0.5	1.11	0.73
G6	2-Propenoic acid, 3-(4-hydroxy-3-methoxyphenyl)-	24.763	1.01	2.7	
S3	Ethanone, 1-(4-hydroxy-3,5-dimethoxyphenyl)-	22.668		0.17	

^a^ DHBF: 2,3-dihydrobenzofuran, H: phenol-type compounds, G: guaiacol-type compounds, S: syringol-type compounds.

Condition: 1 g lignin, 2 mmol ZnCl2, 0.2 g 5 wt% Ru/C, 20 ml methanol, 4 g formic acid, 30 min.

#### 3.1.4 Effect of reaction temperature and time

**FIGURE 3:**
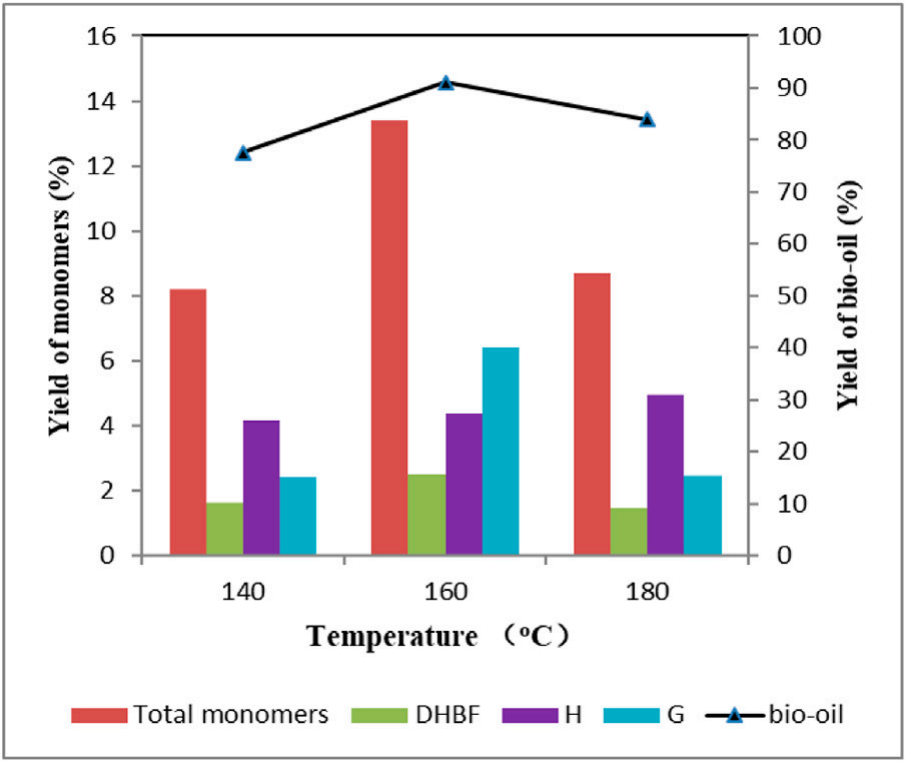
Effect of reaction temperature on the yield of depolymerized products Condition: 1 g lignin, 2 mmol ZnCl_2_, 0.2 g 5 wt% Ru/C, 20 ml methanol, 4 g formic acid, 30 min.

The effect of reaction temperature on lignin depolymerization was investigated (Figure 3). It could be found that lignin depolymerization was highly dependent on temperature. Yields of bio-oil and total aromatic monomers were sharply increased with the elevation of reaction temperature. Remarkably, a noticeable rise was exhibited in the temperature range of 140–160°C. Wherein, yield of bio-oil increased from 77.6 wt% to 91.1 wt%, and yield of total aromatic monomers increased from 8.22 wt% to 13.4 wt%. This indicated that a higher reaction temperature seemed to have a positive influence on lignin depolymerization. However, further increasing the temperature to 180°C, yields of bio-oil and total aromatic monomers decreased to 83.9 wt% and 8.69 wt%, where repolymerization of monomers and oligomers was considered to be responsible for this (Long et al., 2014). As can be seen in Table 4, no other S-type phenolic compounds were produced except for S3 at 160°C. The yield of DHBF first increased to 2.48 wt% and then decreased to 1.45 wt% as a function of temperature. It should be noted that yield of H-type phenolic compounds increased with the elevated reaction temperature, and the highest yield (4.97 wt%) appeared at 180°C. The yield of total G-type phenolic compounds showed a similar tendency with that of bio-oil and total aromatic monomers when reaction temperature rose. One more interesting phenomenon, G4 could be detected only reaction temperature was 160°C. Product distribution showed excessive high temperature promoted the production of H-type compounds, but inhibited G-type compounds, aggravating the repolymerization (Lin et al., 2015). An appropriate temperature is adopted to optimize the yield of product.

**FIGURE 4:**
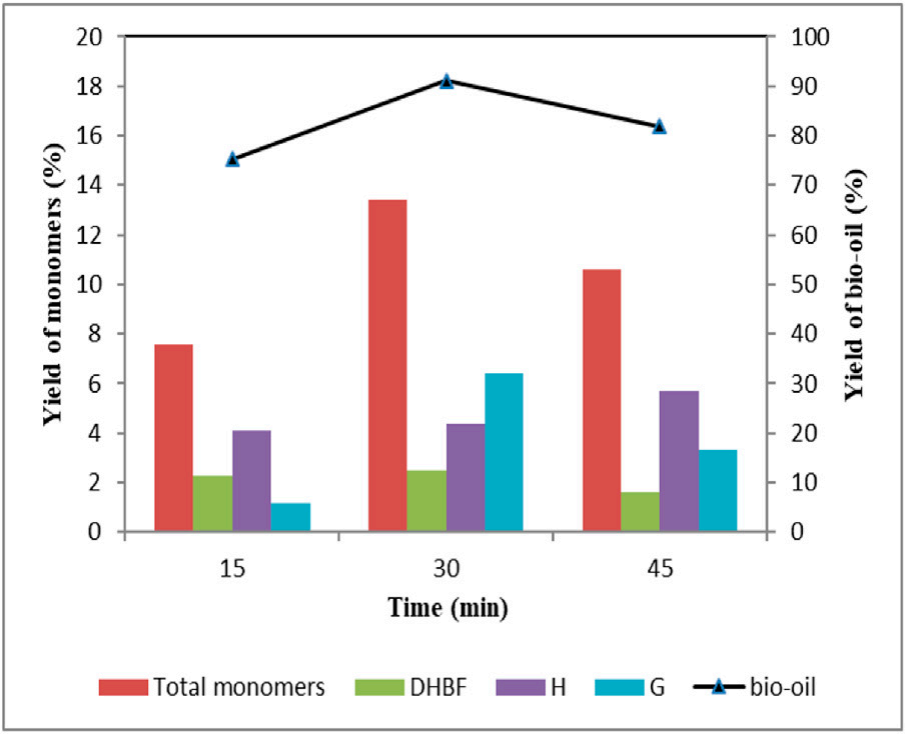
Effect of reaction time on the yield of depolymerized products Condition: 1 g lignin, 2 mmol ZnCl_2_, 0.2 g 5 wt% Ru/C, 20 ml methanol, 4 g formic acid, 160°C.

Figure 4 shows the effect of reaction time on lignin depolymerization, and product distribution is shown in Table 5. As reaction time increased from 15 min to 30 min, bio-oil yield increased from 75.3 wt% to 91.1 wt%, and the total aromatic monomers yield reached 13.4 wt%. Further prolonging the reaction time to 45 min, significant decrease occurred in bio-oil and total aromatic monomers yield, which were 81.8 wt% and 10.6 wt% respectively. Data in Table 5 showed that there were fewer kinds of aromatic monomers when reaction time was 15 min. DHBF and H-type compounds were the major products, which yields were 2.28 wt% and 3.87 wt% respectively. These indicated that short reaction time led to insufficient bond rupture. For G-type compounds, the total yield first increased and then decreased as reaction time increased from 15 min to 45 min. It should be noted that yields of G1 and G2 had been rising with the increase of time. However, other G-type compounds appeared in the products only when the reaction time reached 30 min, which decreased in the case of prolonging the reaction time. These results illustrated that appropriate reaction time was favorable for lignin depolymerization. Short reaction time would make the reaction incomplete. Repolymerization of lignin-derived intermediates was promoted at long reaction time (Miller et al., 2002; Toledano et al., 2014; Shu et al., 2016a). Therefore, it is necessary to determine the ideal reaction time for the efficient depolymerization of lignin.

**TABLE 5:** Effect of reaction time on the distribution of lignin-derived aromatic monomers.

Type^a^	Compound	Retention Time (min)	Yield (%)		
			Reaction time (min)		
			15	30	45
DHBF	Benzofuran, 2,3-dihydro-	13.832	2.28	2.48	1.61
H1	Phenol	8.731	1.2	1.26	1.64
H2	Phenol, 4-ethyl-	12.753	2.32	2.47	3.53
H3	2-Propenoic acid, 3-(4-hydroxyphenyl)-, methyl ester	22.837	0.35	0.66	0.53
G1	Phenol, 2-methoxy-	11.109	0.69	0.91	1.27
G2	Phenol, 4-ethyl-2-methoxy-	14.999	0.5	0.5	0.78
G3	Phenol, 2-methoxy-4-vinyl-	15.679		0.66	0.16
G4	Vanillin	17.293		0.52	0.45
G5	2-Propenoic acid, 3-(4-hydroxy-3-methoxyphenyl)-, methyl ester	24.544		1.11	0.66
G6	2-Propenoic acid,3-(4-hydroxy-3-methoxyphenyl)-	24.763		2.7	
S3	Ethanone, 1-(4-hydroxy-3,5-dimethoxyphenyl)-	22.668		0.17	

^a^ DHBF: 2,3-dihydrobenzofuran, H: phenol-type compounds, G: guaiacol-type compounds, S: syringol-type compounds.

Condition: 1 g lignin, 2 mmol ZnCl_2_, 0.2 g 5 wt% Ru/C, 20 ml methanol, 4 g formic acid, 160°C.

### 3.2 Possible catalytic mechanism

**FIGURE 5:**
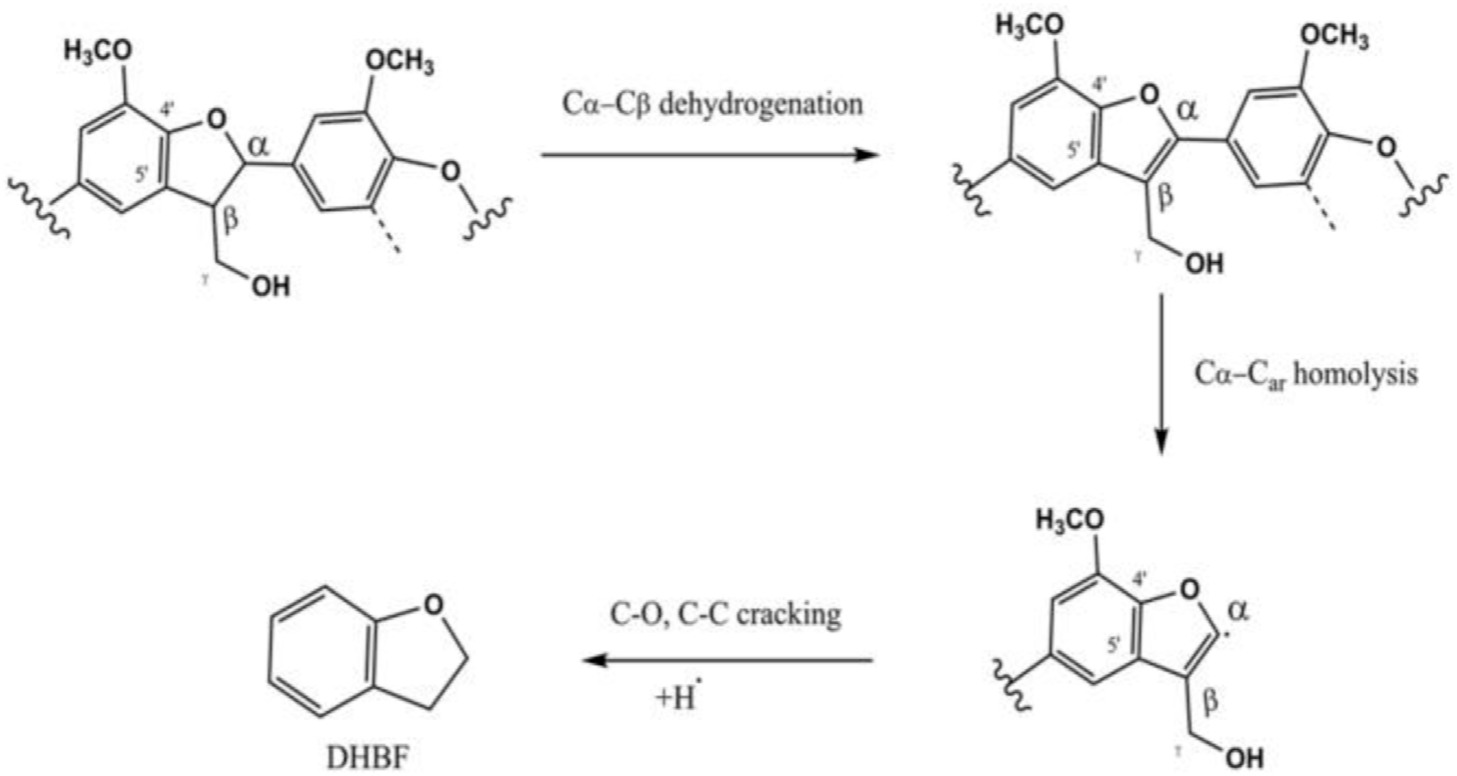
Possible mechanisms of DHBF production.

**FIGURE 6:**
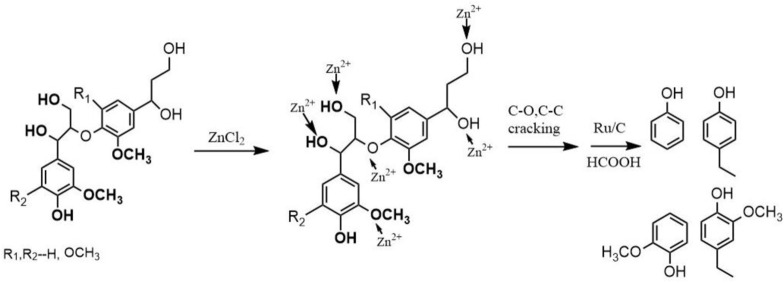
Proposed catalytic mechanism for lignin depolymerization over Ru/C and ZnCl_2_ catalyst.

According to the 2D HSQC NMR spectrum of lignin reported in our previous research (Shao et al., 2018), a large number of β-O-4′ and phenylcoumarin structure existed in the lignin structure (Supplementary Figures S1, S2). During lignin depolymerization, the cleavage of C-O and C-C bonds was realized, producing the aromatic monomers and oligomers. DHBF was mainly produced through breaking C_α_-C_ar_ bond of phenylcoumaran structure. The possible mechanism of DHBF formation was proposed (Figure 5). It should be noted that the fracture of C_α_-C_ar_ bond and ring-opening of phenylfuran ring could not carried out simultaneously according to density functional theory. The result demonstrated yield of lignin-derived bio-oil and aromatic monomers, especially the alkyl phenol and alkyl guaiacol, was effectively improved in the presence of Ru/C and ZnCl_2_. The possible mechanism of lignin depolymerization under the synergetic catalysis of Ru/C and ZnCl_2_ was proposed (Figure 6). A stable complex of Zn^2+^ coordinated to the oxygen atoms in lignin molecule was formed (Klein et al., 2016; Wang et al., 2016). Synchronously, the high electronegativity Cl^−^ was attached to the lignin molecule, selectively weakening the C-O bonds. Afterwards, the cleavage of C-O bonds (including β-O-4 and -OCH_3_ bonds) and C-C bonds occurred under the synergic of Ru/C and the polarization effect of Cl^−^ (Parsell et al., 2013). Finally, various phenols containing alkyl substituents were obtained through a series of typical reactions, such as hydrogenation, dehydration, hydrolysis. Thus, the product distribution can be efficiently controlled by adjusting the type and content of catalyst, reaction temperature, and reaction time.

## 4 Conclusion

Alkali lignin could be efficiently depolymerized by the synergetic catalysis of Ru/C and ZnCl_2_ under microwave heating. 91.1 wt% yield of bio-oil including 13.4 wt% monomers was obtained under the optimum condition (FA-to-lignin mass ratio of 4, Ru/C dosage of 0.2 g, ZnCl_2_ dosage of 2 mmol, 160°C, and 30 min). Formic acid played two roles: (1) acid-catalyzed cleavage of linkages; (2) acted as *in situ* hydrogen donor for hydrodeoxygenation in the presence of Ru/C. This research provided a valuable reference to degrade lignin efficiently and adjust the product distribution under microwave heating.

## Data Availability

The original contributions presented in the study are included in the article/Supplementary Material; further inquiries can be directed to the corresponding authors.
