# Supplementary material for: Efficient depolymerization of lignin through microwave-assisted Ru/C catalyst cooperated with metal chloride in methanol/formic acid media
Source: Front Bioeng Biotechnol. 2022 Dec 16;10:1082341. doi: 10.3389/fbioe.2022.1082341 (PMC9800509; doi:10.3389/fbioe.2022.1082341)
Supplement: Supplementary file 1 [file DataSheet1.docx]

Supplementary Material

**TABLE S1.** The effect of metal chloride on the distribution of lignin-derived aromatic monomers

| **Type ^b^** | **Compound** | **Retention time**  **（min）** | **Yield(%)** | | | | | | | | | | |
| --- | --- | --- | --- | --- | --- | --- | --- | --- | --- | --- | --- | --- | --- |
|  |  |  | **Metal chloride** | | | | | | | | | | |
|  |  |  | **_^a^** | **AlCl_3_** | **ZnCl_2_** | **CrCl_3_** | **LiCl** | **FeCl_3_** | **NaCl** | **KCl** | **FeCl_2_** | **MgCl_2_** | **Zn(OAC)_2_** |
| DHBF | 2,3-Dihydrobenzofuran | 13.832 | 2.57 | 1.96 | 5.58 | 4.41 | 2.34 | 2.32 | 2.45 | 2.34 | 2.36 | 3.07 | 2.68 |
| H2 | Phenol, 4-ethyl- | 12.753 |  |  | 0.89 |  | 0.06 |  | 0.07 | 0.05 | 0.32 | 0.09 | 0.09 |
| H3 | 2-Propenoic acid, 3-(4-ydroxyphenyl)-, methyl ester | 22.837 | 0.12 | 2.11 | 0.44 | 0.28 | 0.08 | 2.34 | 0.09 | 0.1 | 0.96 | 0.35 | 0.29 |
| H4 | *p*-Coumaric acid | 23.356 | 1.18 |  |  | 1.55 | 0.59 | 0.6 | 0.94 | 0.98 | 1.27 | 1.03 | 1.1 |
| G2 | Phenol, 4-ethyl-2-methoxy- | 14.999 |  |  | 0.25 |  |  |  |  |  | 0.14 |  | 0.05 |
| G3 | Phenol, 2-methoxy-4-vinyl- | 15.679 |  | 0.87 | 0.98 | 1.2 | 0.62 | 0.74 | 0.72 | 0.72 | 0.36 | 1.14 | 0.68 |
| G4 | Vanillin | 17.293 | 0.23 | 0.31 | 0.33 | 0.33 | 0.22 | 0.28 | 0.27 | 0.24 | 0.25 | 0.39 | 0.26 |
| G5 | 2-Propenoic acid, 3-(4-hydroxy-3-methoxyphenyl)-, methyl ester | 24.544 | 0.47 |  | 0.85 | 0.62 | 0.42 | 0.53 | 0.53 | 0.49 | 0.67 | 0.56 | 0.41 |
| G6 | 2-Propenoic acid,3-(4-hydroxy-3-methoxyphenyl)- | 24.763 | 0.37 |  | 0.6 |  | 0.3 | 0.35 | 0.4 | 0.14 | 0.33 | 0.4 | 0.5 |
| S1 | Benzaldehyde, 4-hydroxy-3,5-dimethoxy- | 21.572 | 0.08 | 0.15 |  | 0.14 | 0.1 | 0.17 | 0.04 | 0.04 | 0.15 | 0.15 | 0.11 |
| S2 | Phenol, 2,6-dimethoxy-4-propenyl- | 22.136 | 0.15 |  |  | 0.25 | 0.11 | 0.08 | 0.24 | 0.22 |  | 0.2 | 0.32 |
| S3 | Ethanone, 1-(4-hydroxy-3,5-dimethoxyphenyl)- | 22.668 | 0.13 |  | 0.24 | 0.14 | 0.08 | 0.12 | 0.09 | 0.11 |  | 0.1 | 0.15 |
| S4 | 3,5-Dimethoxy-4-hydroxycinnamaldehyde | 26.137 | 0.08 |  |  | 0.11 | 0.05 |  |  | 0.04 |  |  | 0.05 |
| ^a^: without metal chloride ^b^: DHBF: 2,3-dihydrobenzofuran, H: phenol-type compounds, G: guaiacol-type compounds, S: syringol-type compounds | | | | | | | | | | | | | |


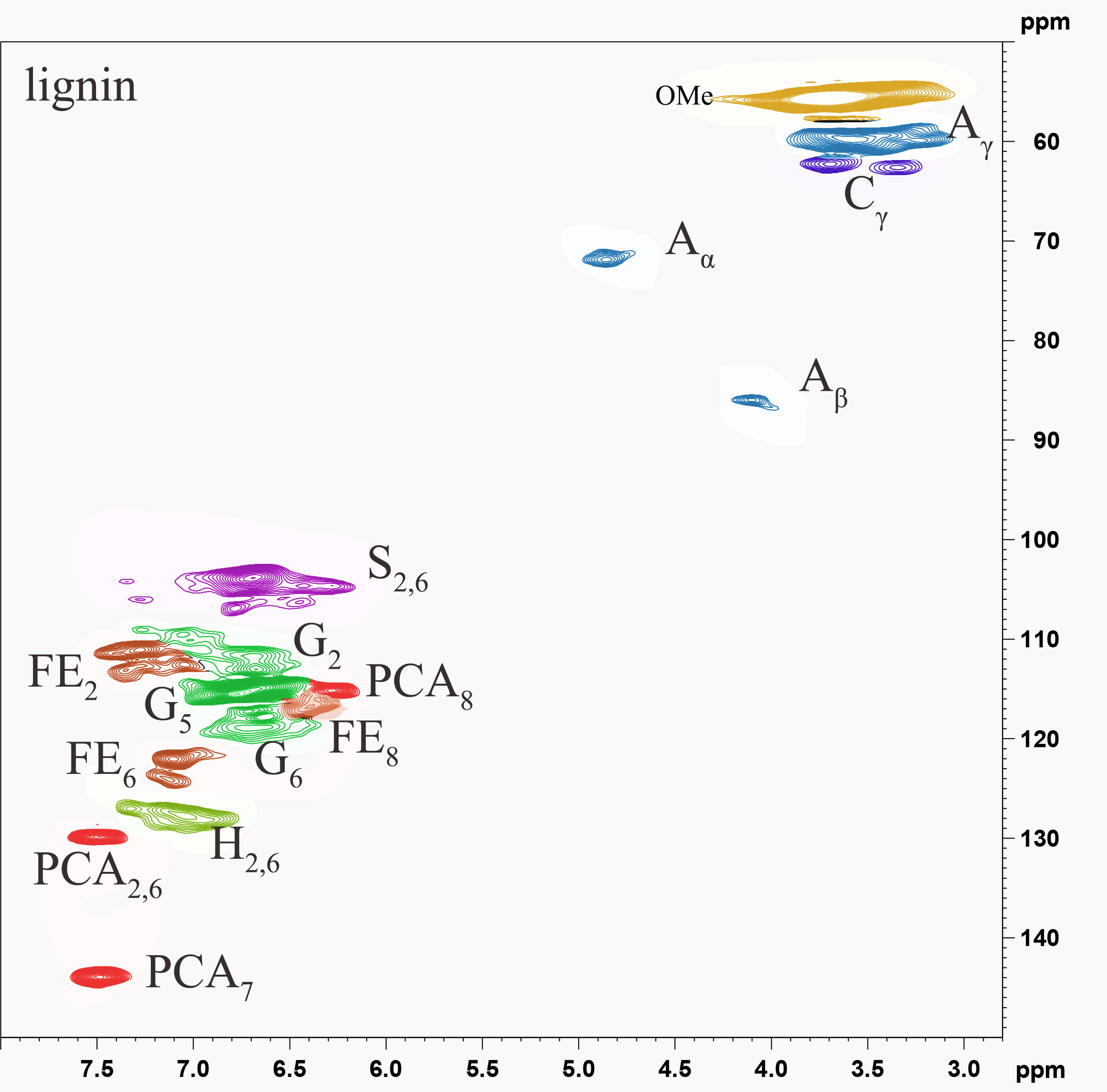


**FIGURE S1** 2D HSQC NMR spectra of lignin


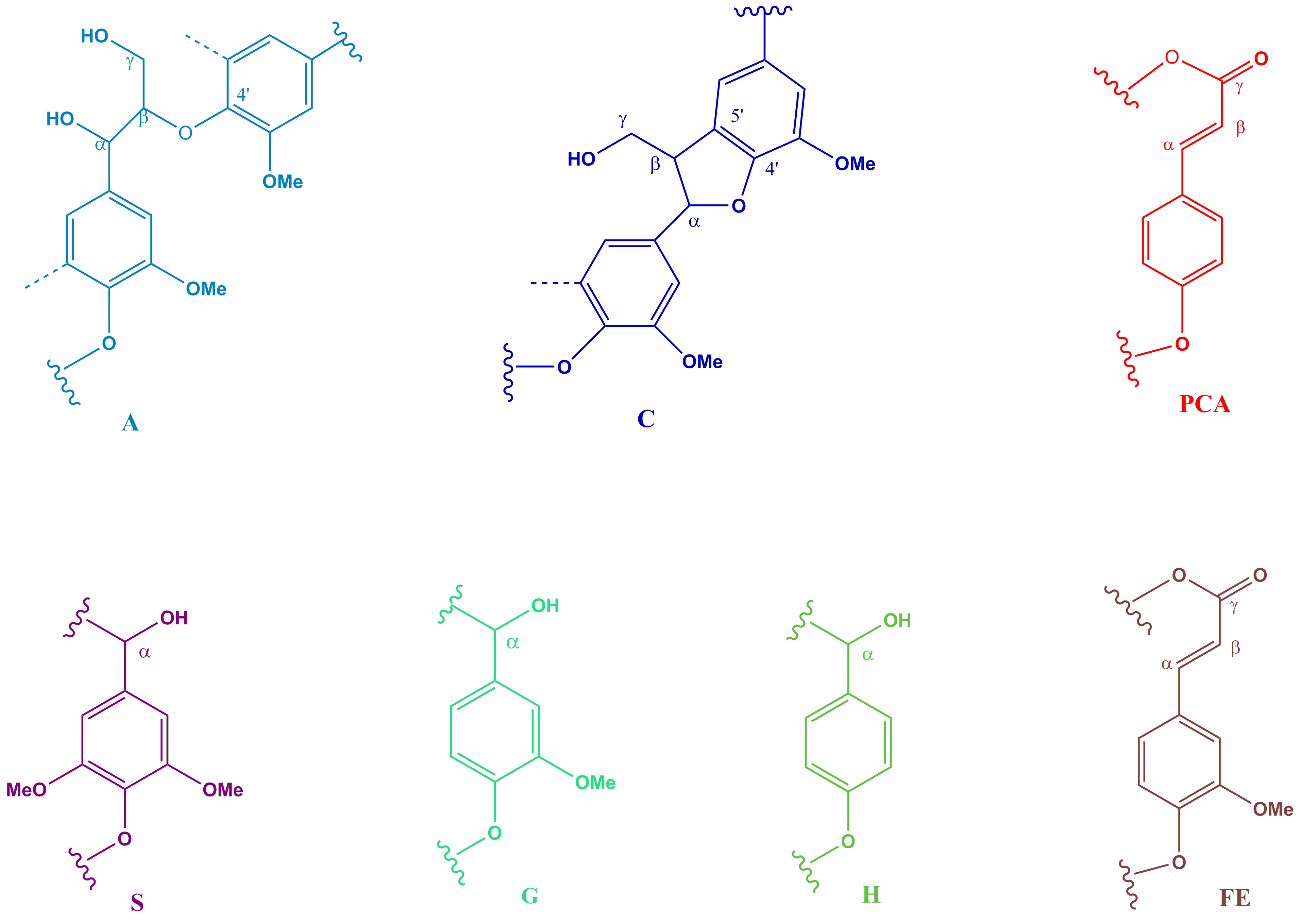


**FIGURE S2** Main classical substructures, involving different side-chain linkages, and aromatic units identified by 2D NMR of the starting lignin and bio-oil: (A) β-O-4' linkages; (C) phenylcoumaran structures; (PCA) *p*-coumarate units; (FE) ferulate units; (S) syringyl units; (G) guaiacyl units; (H) *p*-hydroxyphenyl units.
